# Torque Teno Virus Primary Infection Kinetics in Early Childhood

**DOI:** 10.3390/v14061277

**Published:** 2022-06-11

**Authors:** Elina Väisänen, Inka Kuisma, Marjaana Mäkinen, Jorma Ilonen, Riitta Veijola, Jorma Toppari, Klaus Hedman, Maria Söderlund-Venermo

**Affiliations:** 1 Department of Virology, University of Helsinki, 00290 Helsinki, Finland; elina.vaisanen@thl.fi (E.V.); inka.kuisma@hologic.com (I.K.); klaus.hedman@helsinki.fi (K.H.); 2 MediCity, University of Turku, 20520 Turku, Finland; marjaana.makinen@utu.fi; 3 Immunogenetics Laboratory, Institute of Biomedicine, University of Turku, 20520 Turku, Finland; jsilonen@utu.fi; 4 PEDEGO Research Unit, Medical Research Center, Department of Pediatrics, Oulu University Hospital and University of Oulu, 90220 Oulu, Finland; riitta.veijola@oulu.fi; 5 Centre for Population Health Research and Research Centre for Integrated Physiology and Pharmacology, Institute of Biomedicine, University of Turku, 20520 Turku, Finland; jortop@utu.fi; 6 Department of Pediatrics, Turku University Hospital, 20520 Turku, Finland; 7 Helsinki University Hospital Laboratory (HUSLAB), 00290 Helsinki, Finland

**Keywords:** anellovirus, TTV primary infection, infants, genoprevalence, viremia

## Abstract

Human torque teno viruses (TTVs) are a diverse group of small nonenveloped viruses with circular, single-stranded DNA genomes. These elusive anelloviruses are harbored in the blood stream of most humans and have thus been considered part of the normal flora. Whether the primary infection as a rule take(s) place before or after birth has been debated. The aim of our study was to determine the time of TTV primary infection and the viral load and strain variations during infancy and follow-up for up to 7 years. TTV DNAs were quantified in serial serum samples from 102 children by a pan-TTV quantitative PCR, and the amplicons from representative time points were cloned and sequenced to disclose the TTV strain diversity. We detected an unequivocal rise in TTV-DNA prevalence, from 39% at 4 months of age to 93% at 2 years; all children but one, 99%, became TTV-DNA positive before age 4 years. The TTV-DNA quantities ranged from 5 × 10^1^ to 4 × 10^7^ copies/mL, both within and between the children. In conclusion, TTV primary infections occur mainly after birth, and increase during the first two years with high intra- and interindividual variation in both DNA quantities and virus strains.

## 1. Introduction

Human torque teno viruses (TTVs) are small nonenveloped viruses with circular, single-stranded DNA genomes belonging to the *Alphatorquevirus* genus in the *Anelloviridae* family [1]. The compact ≤ 4-kb TTV genomes all have a similar genome organization, with a short conserved untranslated region (UTR), but an extraordinarily diverse coding area [1,2,3].

TTV causes persistent viremia, and is very prevalent across different populations worldwide: by sensitive PCR methods, targeting the conserved regions, over 90% of adults have TTV DNA in their blood at any given time point [2,4,5]. To date, no causative roles in diseases have been identified, although the TTV DNA loads among diseased or immunocompromised individuals seem to be higher than in healthy individuals [6,7]. One individual can also have several different TTV strains simultaneously circulating in the blood [3,8,9,10]. TTVs could thus be considered as part of the human microbiome [2,3,11].

TTV DNA has been detected in practically all organs and secretions of the body; however, the routes of infection are not well known. It has further been debated whether newborn babies are already infected in utero. Nevertheless, one recent study found no TTV in umbilical cord blood [12]; others have shown the TTV prevalence to grow with age [13,14,15].

In the present study, we screened sequential serum samples from 102 healthy infants/children to further pinpoint the time of primary infection and to measure the viral loads. In addition, we analyzed all 9 to 18 samples available from each of a group of 18 children to elucidate the variations in TTV DNA quantity over time. Lastly, the TTV PCR amplicons in a subset of samples from 5 of these 18 children were cloned and sequenced to give an overview of the TTV strains and their diversity.

## 2. Materials and Methods

### 2.1. Study Subjects and Samples

The participants were part of the population-based type 1 diabetes prediction and prevention (DIPP) study in Finland, which is a birth cohort study monitoring clinical events preceding type 1 diabetes in children with HLA-associated genetic susceptibility to the disease [16,17,18]. Every child was followed up since infancy, at intervals of ~3 months, until 2 years of age and, subsequently, of ~6 months until 15 years of age. Altogether 102 children were included in the present study, based on the availability of samples and participant age (at first sampling: mean 3.7 mo, median 3.4, range 2.4–6.8). All children were healthy at the sampling times, and none progressed to clinical diabetes.

The TTV DNA was analyzed by quantitative (q)PCR starting from the first two consecutive serum samples of each child in parallel, and if negative, the testing was continued with the next two samples, etc., until reaching the first TTV-DNA-positive sample(s). In total, 474 samples were analyzed, 2 to 18 per child (median 2, mean 4.6) depending on the TTV DNA conversion time. To investigate the potential fluctuation in TTV viremia, all available samples were analyzed from 18 children (*n* = 213; median 11 per child, range 9–18; median follow-up time 57 months, range 45–83). These 18 children were selected based on the maximal amount of follow-up samples available and included the four children who were followed the longest before converting, as well as those who converted early.

### 2.2. Ethics

The ethics committee of the Hospital District of Southwest Finland approved the study. The legal guardians of the participants provided written informed consent, and the study, including sampling, was conducted in accordance with relevant guidelines and regulations.

### 2.3. DNA Extraction

Due to serum scarcity, the DNA was extracted by phenol-chloroform from 30 µL of serum, precipitated by sodium acetate and ethanol, and the DNA was resuspended into 30 μL of TE buffer (10 mM Tris, 0.1 mM EDTA; pH 8.0). A positive-DNA-extraction control, 30 µL of serum with a known low TTV load (the same serum throughout the study), and a negative control (H_2_O) were included in each DNA extraction round.

### 2.4. TTV Quantification

The TTV qPCR was performed as described [19] with primers and probes presented in Table 1. It amplifies a 63-nt product from the most conserved UTR region and recognizes essentially all TTV strains. A ten-fold dilution series of plasmid TTV 10B (GenBank #MT448658) served as the positive qPCR control and standard. Water was included as negative qPCR controls; one on each qPCR strip, i.e., every seventh well was a negative control. Every sample was analyzed in duplicate, with 5 µL of DNA per well. The sample was considered positive when either replicate was positive. Such an interpretation was followed for several reasons: (i) the quantity of TTV is often low and close to the assay’s detection threshold (of note, all samples with a single positive replicate were of very low copy numbers); (ii) divergent TTV types may be amplified less efficiently; and (iii) all negative controls throughout the study remained negative, ruling against contamination. In addition, strict precautions to avoid contamination were taken; sample handling, master mix preparations, and amplifications were all carried out in separate rooms with protective clothing and single-use disposable materials and filtered tips.

### 2.5. TTV Genotyping

#### 2.5.1. Sample Selection for TTV Genotyping

Based on the qPCR results, samples from 5 of the 18 children, of whom all available samples were analyzed, were selected for TTV DNA sequencing and genotyping. The amplicons from 17 samples (3–4 per child) were sequenced and analyzed, 5 clones per sample, to reveal diversity.

#### 2.5.2. Rolling Circle Amplification

To facilitate the amplification of a longer TTV amplicon in samples with low virus quantity as well as to enable the amplification of potential minor TTV populations, the samples were preamplified with randomly primed rolling circle amplification (RCA). The 25-µL RCA reactions consisted of 10 mM of each dNTP, 20 µM exo-resistant random primer (Fermentas, Vilnius, Lithuania), 5 U of phi29 DNA polymerase (Fermentas), 1× phi29 DNA polymerase reaction buffer (Fermentas) and 5 µL of DNA template. The reactions were carried out at 30 °C for 18 h followed by inactivation by heating at 65 °C for 10 min.

#### 2.5.3. Multiplex PCR for TTV Typing

For TTV typing, a multiplex PCR was designed, amplifying a ~500-nt region. The amplicons comprised the ORF2 and the near-full-length conserved UTR regions, and their lengths were between 430 nt and 570 nt depending on the TTV strain and which of the multiple reverse primers the amplification used. All primers are listed in Table 1. The multiplex PCR reactions consisted of 2.5 U of AmpliTaq Gold DNA Polymerase (Applied Biosystems, Foster City, CA, USA); 1× PCR buffer II (Applied Biosystems); 5% DMSO; 0.5 µM of forward primer UTR5; 0.8 µM of reverse primer 9 rU; 0.4 µM each of reverse primers 9r1, 9r11, 9r20, 9r21, 9r23, 9r46, 12r, 24r, 25r, and 27r; 3 µL of inactivated RCA reaction as template; and molecular-biology-grade H_2_O to a final volume of 50 µL. The initial denaturation and enzyme activation, at 95 °C for 10 min, was followed by 40 cycles of amplification, consisting of 40 s at 95 °C, 75 s at 53 °C, and 45 s at 72 °C, and a final extension of 5 min at 72 °C.

#### 2.5.4. Cloning and Sequencing

All multiplex PCR products were purified with High Pure PCR Product Purification Kit (Roche, Mannheim, Germany) and cloned into pSTBlue-1 AccepTor™ Vector (Novagen, Madison, WI, USA). At least 5 individual clones per sample were sequenced to detect possible coinfections of different TTV strains.

#### 2.5.5. TTV Control Plasmids for TTV Genotyping

To set up and optimize the TTV-genotyping multiplex PCR, we used plasmids HEL32 (GenBank #AY666122), 2h (#AY823988), 3h (#AY823989), and 10B (#MT448658). In addition, 14 different TTV strains were cloned directly from the sera of healthy adults. The viral DNAs were amplified with primers UTR5 and p1 or p1gr4 (Table 1) resulting in 1.0–1.2 kb pieces of different TTV DNAs, which were cloned into pSTBlue (Novagen). Of note, 5 of the 14 cloned TTV strains belonged to genogroup 4, and all 5 had a point mutation in the TTV qPCR reverse-primer (AMTAS rev) area. The addition of AMTAS gr4 rev primer corrects this mismatch and enhances the qPCR performance. The obtained sequences were submitted to GenBank (accession numbers ON323527-ON323540).

## 3. Results

### 3.1. TTV Prevalence and Quantity in Children

**Figure 1:**
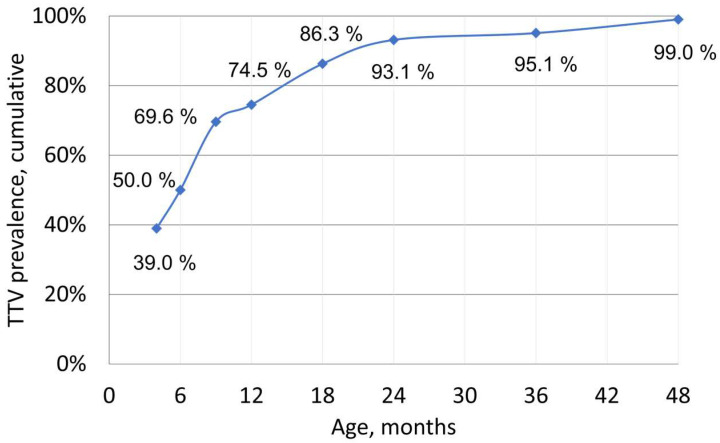
Cumulative TTV DNA prevalence in children. The time points (4, 6, 9, 12, 18, 24, 36, and 48 months of age) depict the percentages of children with TTV DNA in one or more samples prior to that age. The first time point was set at <4 months instead of <3 months as there were too few samples from infants <3 mo of age. At the <4-month time point, samples from 77 children were available, of which 30/77 (39%) were TTV-DNA-positive. At the <6-month time point, samples from 98 children were available and 49/98 (50%) of the children had become TTV-DNA-positive. For the other time points, the curve depicts the cumulative TTV prevalence of the entire cohort comprising 102 children.

An indisputable rise in TTV prevalence with age was observed: TTV DNA was harbored by 39.0% of the <4-month-olds, compared to 50% in 6-month-olds, 74.5% of the <12-month-olds, and 93.1% of the <24-month-olds (Figure 1). Altogether, among the 102 children, 101 (99%) became TTV-DNA-positive by age 4. The single permanently TTV-DNA-negative child was followed until age 4 with nine samples (child #175). The background and infectious history of this child did not reveal any particular differences to those of the other children.

**Figure 2:**
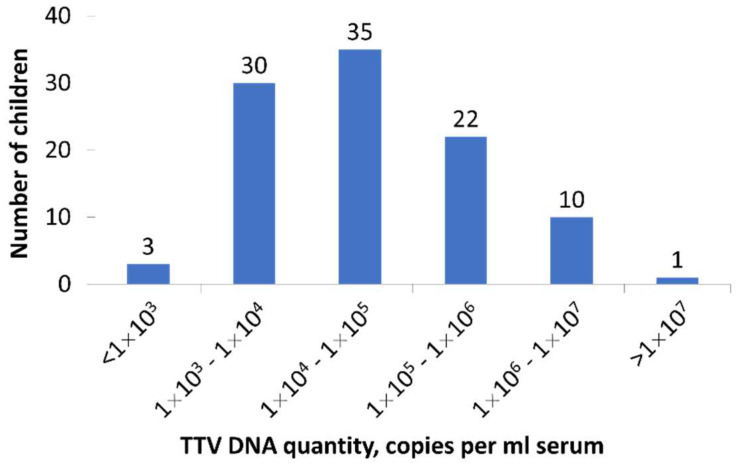
The histogram shows the distribution of TTV viral loads in the first TTV-DNA-positive sample of each child (*n* = 101 children). The numbers above columns represent numbers of children.

The viral load in the first TTV-DNA-positive sample of each child ranged from 6 × 10^1^ to 1.8 × 10^7^ copies per ml of serum (median 3.5 × 10^4^). Overall, the TTV quantities during the presumed primary infections were not very high. Only 11/101 (10.9%) children had more than 1 × 10^6^ copies per ml serum, and in most cases (68/101 (67.3%)) the viral quantities were below 1 × 10^5^ (Figure 2).

For ten children, in the first TTV-DNA-positive sample only one of the two qPCR replicates was positive, and the viral loads were very close to the detection threshold. In each case, the TTV DNA quantity was lower than 22 copies per reaction (range 2.7–22 copies per reaction, median 9.6), corresponding to 4.4 × 10^3^ copies per ml serum. The interpretation that a child was TTV-positive with just a single positive replicate was supported by the fact that among 9 of the 10 children, the subsequent sample (taken on average 3.8 months [range 2.6–5.9] later) showed, in both replicates, a higher TTV DNA level (range 7.1 × 10^3^ – 4 × 10^7^ copies per ml serum, median 1.2 × 10^5^).

The TTV DNA quantities, in the 248 TTV-positive samples, were observed to fluctuate, both within each child and between children (range 5 × 10^1^–4 × 10^7^ per ml serum, median 4 × 10^4^). The highest copy number detected was 4 × 10^7^, and overall, only 5/248 (2.0%) samples harbored more than 1 × 10^7^. There were no striking differences in the background or infection history of these five children compared to the other children. On the other hand, 65.2% (162/248) of the samples containing TTV DNA had fewer than 1 × 10^5^ copies per ml.

The TTV DNA extraction control was consistently qPCR-positive, with comparable quantities (range 1.4 × 10^3^ to 4.8 × 10^4^ per ml serum (median 1.7 × 10^4^, mean 1.8 × 10^4^) in 42 extractions). All the negative controls (DNA extraction; qPCR) remained negative.

### 3.2. TTV Quantity Follow-Up

**Figure 3:**
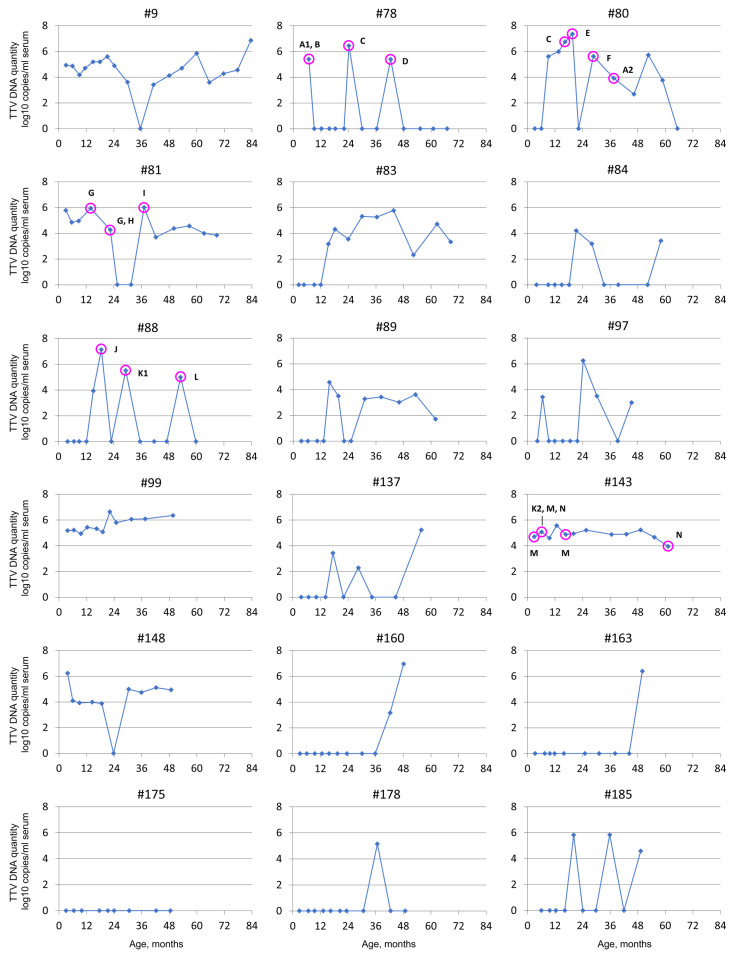
Serum TTV DNA quantity fluctuations in children (*n* = 18) followed up for 4 to 7 years. The TTV strains present in a total of 17 selected samples (indicated with purple rings) of five children were further analyzed with multiplex PCR and sequencing. The different TTV strains are labeled with different letters to facilitate easy differentiation of the types in the figure. NB, the letters are in alphabetical order and do not correlate with any ICTV or other classifications. A1 and A2 types are, on the nucleotide level, 95.2–96.1% similar to each other, and K1 and K2 are 95.0–95.2% similar. Type N is 90.5–90.9% similar to K1 and 88.3–88.6% similar to K2, and therefore defined as a different strain. Other strains are 75% or less similar to each other. See Figure 4, phylogenetic tree, for more information.

Highly variable patterns in viral load were observed among the 18 children followed through the full sampling period (Figure 3). Only one child (#175) remained thoroughly TTV-DNA-negative and three (#160, #163, #178) acquired the viral DNA very late, at 37 to 50 months of age. Of the 14 TTV-DNA-positive children selected for longer follow-ups, 8 (#9, #80, #81, #83, #89, #99, #143, and #148) remained mainly positive throughout the follow-up, with 2 of them (#99 and #143) being positive in every single sample, and 1 (#83) in every sample after conversion at 1 year of age. Only two (#99 and #143) had relatively stable TTV quantities, whereas ten children (#9, #78, #80, #81, #83, #88, #97, #137, #148, and #185) showed extreme fluctuations (Figure 3).

### 3.3. TTV Strains in Follow-Up

**Figure 4:**
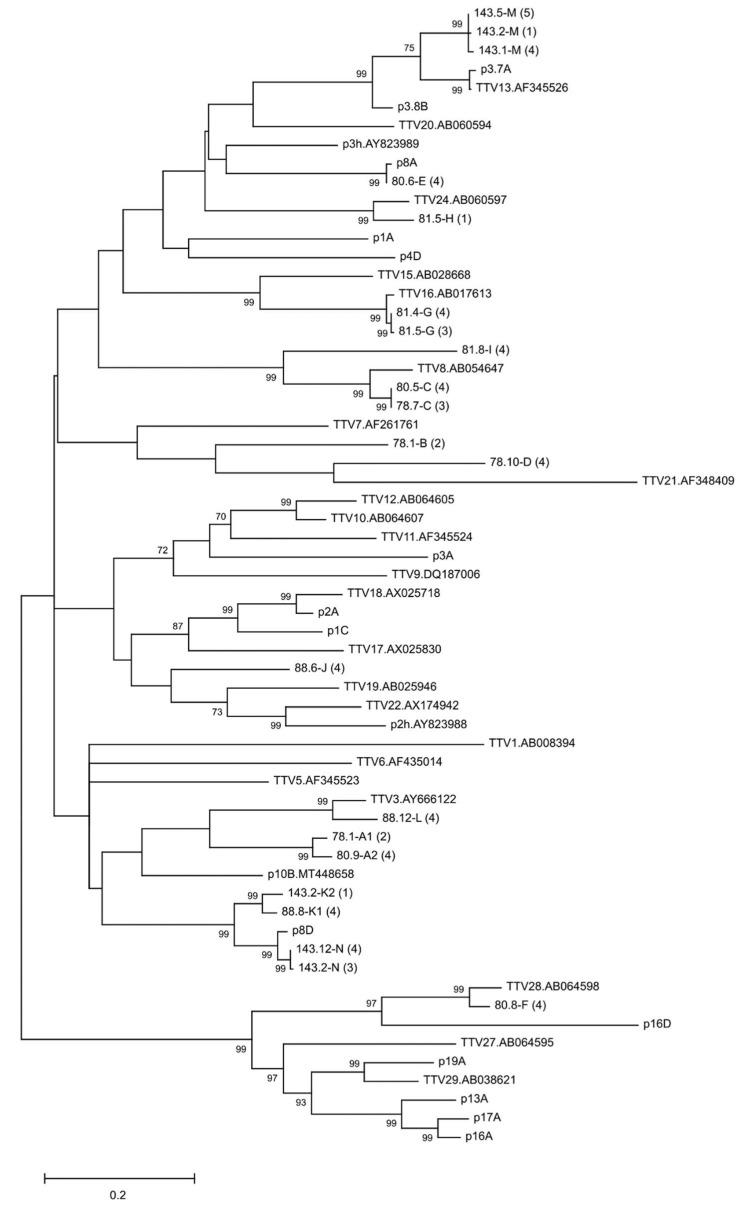
Phylogenetic tree of different TTV types. The tree includes (i) cloned and sequenced TTVs from the children (the number in parentheses depicts how many clones from the sample had the same sequence), (ii) all TTV-type species detected in humans, and (iii) all TTV clones/plasmids used in this study as controls for method development (marked as pXXX in the tree; see also Section 2.5.5). GenBank number of the previously published sequences is mentioned on the tree. The tree is made based on the ~500 nt region of ranging from the conserved UTR to close to the end of ORF2 (the multiplex PCR amplicon area). The phylogenetic tree was generated in MEGA X using the maximum likelihood method and general time reversible model, and bootstrap values were determined by 1000 replicates. Branch support values are given when >70.

The TTV strains in 3–4 samples each of five early-converting children that were followed longer were genotyped (magenta circles depicted in Figure 3). In each of these children, the TTV strain changed over time, and in 3 samples out of 17, more than one TTV strain was present, confirming that coinfections of different TTV strains are common (Figure 3, children #78, #81, and #143). Only two children maintained, in more than one sample, any given TTV strain: in child #81, strain “G” in two consecutive samples taken 8.5 months apart; in child #143, strain “M” in three samples taken at 3, 6, and 16 months of age; and strain “N” in two samples taken at 6 and 61 months of age (Figure 3 and Figure 4). The resulting sequences were submitted to GenBank (accession numbers ON423621-ON423689).

## 4. Discussion

TTV has been known for nearly 25 years; however, these highly divergent viruses still remain enigmatic. Nearly all adults at any given time have TTV(s) circulating in their blood, and while antibodies continuously clear the virus, de novo replication maintains the chronic viremia. No disease association has been established, although elevated viral loads have been observed, e.g., in children with respiratory illness [20]. Whether, or to what extent, the primary infection takes place already in utero is controversial [21,22,23]. Technical challenges are exemplified by suboptimal PCRs and contamination-prone umbilical cord sampling. However, in a study utilizing sensitive methods, no TTV DNA was detected in the umbilical cord blood of 84 newborns of TTV-DNA-positive mothers [12].

Rather than umbilical cord blood, we thus examined by qPCR 474 follow-up serum samples of 102 children from 2.5 months up to 7 years of age to disclose the time of TTV primary infection, the corresponding viral loads, and their kinetics. A subset of samples was further analyzed for the infecting TTV strains. A rapid increase in TTV prevalence was observed during the first year of life. Consequently, it appears likely that TTV primary infections indeed generally occur after birth rather than in utero [12,13,14].

All children except one became TTV-DNA-positive before age 4, substantiating TTV’s ubiquity. In a third of the serum specimens, the TTV DNA loads were low, whereas they were high in only every tenth. Our qPCR assay targets the most conserved region of the TTV genome and is highly sensitive. In addition, we had an extra reverse primer to ensure the amplification of also more diverse TTVs, particularly those of genogroup 4. Designed for prevalence studies, our method finds even low viral loads and variable strains.

Overall, the loads in the first genopositive samples were rather low, perhaps due to these children’s young age and good health. Indeed, only ~11% of them had more than 1 × 10^6^ TTV copies per ml serum. However, the observed viral loads (median 3.5 × 10^4^ per ml of serum) were very similar to those reported previously in 0–12-month-old children by Tyschik et al. (median 5 × 10^4^ per ml of whole blood) [13]. The highest TTV DNA serum level among the 474 sera of our 102 children was 4 × 10^7^ per ml. When comparing the symptoms of the children with the highest TTV loads with those of the other children, no differences were observed.

The 18 children followed exceptionally long for TTV loads and strains exhibited highly variable virus kinetics and strain profiles. Since the initial genoconversion, eight children maintained the viral DNA in most of their samples, and three in every sample. Conversely, in ten children, the TTV loads fluctuated strongly, at times even falling below the detection limit. Three other children acquired TTV DNA very late, after age 3, and one child remained negative throughout the observation of 4 years. This emphasizes the high intra- and interindividual variation in TTV loads and kinetics. TTV loads have been shown to increase with, e.g., severity of immunosuppression or other virus infections [7,20,24]. However, no such reasons for fluctuation could be found in these children.

Upon TTV genotyping, depicted in Figure 3 and Figure 4, we encountered in 17 samples altogether 16 different TTV strains. While most of the genopositive sera harbored a single TTV strain, some had two or three. The strains also fluctuated with time, and they could differ in two consecutive samples taken only 3 months apart. Persistence of a given TTV strain was observed in two out of five children. The TTV “N” sequences were 98.8–99.2% similar in the two samples taken 4.5 years apart, demonstrating that an individual can harbor a given TTV type over several years either via persistence or repeated infection(s). Interestingly, only one TTV type (“C”) and two closely related strains (A1 and A2; K1 and K2) were observed in more than one child (Figure 3).

## 5. Conclusions

In summary, our results show that TTV generally appeared in children after birth, with the overall TTV prevalence reaching 93% by the second birthday. In addition, the TTV strain(s) in young children could either change rapidly or persist for years, with stable or fluctuating loads. The mechanism and significance of this large and diverse variation in both TTV strains and DNA levels awaits elucidation.

## Figures and Tables

**Table 1:** Oligos used in the study.

Method	Oligo Name	Sequence 5′ ⟶ 3′
TTV qPCR	AMTS fwd	GTGCCGNAGGTGAGTTTA
	AMTAS rev	AGCCCGGCCAGTCC
	AMTAS gr4 rev	AGCCCGGCCAGACC
	AMTPTU probe	FAM-GGGGCAATTCGGGCT-BHQ1
TTV multiplex PCR for ~500 nt amplicon	UTR5	GGGTGCCGRAGGTGAGTTTAC
	9rU	CGSCGYCTCCTTACTSTTC
	9r1	CGGCGTCTCCTTACGTTTC
	9r11	CCGYCTACTCACATATCGTC
	12r	AACAGCTCGTCGAGKTCTTC
	9r20	CTSCGCCTCCTTACTCGTC
	9r21	CCTCCGYCTCCTTACTSTG
	9r23	CCTGCKCCTCCTTACTSTTG
	24r	CGTCTATAGCGTCGAGSAGGTC
	25r	GTCTCCTTACTCTGGGKCGTCTA
	27r	AGCTCTTCGTCTGCGAGKTCT
	9r46	CATCTCCTACTTACAWATCGTC
Rev primers for amplifying TTV control plasmids	p1	GTTWGTGGTGRGCHGAAYGG
	p1gr4	CCACTTGTTTAGCATKAGTTTDGG

N = A, C, G or T; R = A or G; Y = C or T; K = G or T; S = C or G; W = A or T.

## Data Availability

The obtained TTV sequences have been submitted to GeneBank. All data and material produced in this study are available from the corresponding author upon request.
